# Glycolytic reprogramming in macrophages and MSCs during inflammation

**DOI:** 10.3389/fimmu.2023.1199751

**Published:** 2023-08-22

**Authors:** Xueping Li, Huaishuang Shen, Mao Zhang, Victoria Teissier, Ejun Elijah Huang, Qi Gao, Masanori Tsubosaka, Masakazu Toya, Junichi Kushioka, Chima V. Maduka, Christopher H. Contag, Simon Kwoon-Ho Chow, Ning Zhang, Stuart B. Goodman

**Affiliations:** ^1^ Department of Orthopaedic Surgery, Stanford University School of Medicine, Stanford, CA, United States; ^2^ Department of Orthopaedic Surgery, First Affiliated Hospital of Soochow University, Suzhou, China; ^3^ Cardiovascular Institute Operations, Stanford University School of Medicine, Stanford, CA, United States; ^4^ Departments of Biomedical Engineering and Microbiology & Molecular Genetics, Michigan State University, East Lansing, MI, United States; ^5^ Institute for Quantitative Health Science and Engineering, Michigan State University, East Lansing, MI, United States; ^6^ Department of Orthopaedics and Traumatology, The Chinese University of Hong Kong, Hong Kong, Hong Kong SAR, China; ^7^ Department of Bioengineering, Stanford University, Stanford, CA, United States

**Keywords:** inflammation, mitochondrial bioenergetics, bone marrow-derived cells, macrophage, mesenchymal stromal cell

## Abstract

**Background:**

Dysregulated inflammation is associated with many skeletal diseases and disorders, such as osteolysis, non-union of fractures, osteonecrosis, osteoarthritis and orthopaedic infections. We previously showed that continuous infusion of lipopolysaccharide (LPS) contaminated polyethylene particles (cPE) caused prolonged inflammation and impaired bone formation. However, the metabolic and bioenergetic processes associated with inflammation of bone are unknown. Mitochondria are highly dynamic organelles that modulate cell metabolism and orchestrate the inflammatory responses that involve both resident and recruited cells. Glycolytic reprogramming, the shift from oxidative phosphorylation (OXPHOS) to glycolysis causes inappropriate cell activation and function, resulting in dysfunctional cellular metabolism. We hypothesized that impaired immunoregulation and bone regeneration from inflammatory states are associated with glycolytic reprogramming and mitochondrial dysfunction in macrophages (Mφ) and mesenchymal stromal cells (MSCs).

**Methods:**

We used the Seahorse XF96 analyzer and real-time qPCR to study the bioenergetics of Mφ and MSCs exposed to cPE. To understand the oxygen consumption rate (OCR), we used Seahorse XF Cell Mito Stress Test Kit with Seahorse XF96 analyzer. Similarly, Seahorse XF Glycolytic Rate Assay Kit was used to detect the extracellular acidification rate (ECAR) and Seahorse XF Real-Time ATP Rate Assay kit was used to detect the real-time ATP production rates from OXPHOS and glycolysis. Real-time qPCR was performed to analyze the gene expression of key enzymes in glycolysis and mitochondrial biogenesis. We further detected the gene expression of proinflammatory cytokines in Mφ and genes related to cell differentiation in MSC during the challenge of cPE.

**Results:**

Our results demonstrated that the oxidative phosphorylation of Mφ exposed to cPE was significantly decreased when compared with the control group. We found reduced basal, maximal and ATP-production coupled respiration rates, and decreased proton leak in Mφ during challenge with cPE. Meanwhile, Mφ showed increased basal glycolysis and proton efflux rates (PER) when exposed to cPE. The percentage (%) of PER from glycolysis was higher in Mφ exposed to cPE, indicating that the contribution of the glycolytic pathway to total extracellular acidification was elevated during the challenge of cPE. In line with the results of OCR and ECAR, we found Mφ during cPE challenge showed higher glycolytic ATP (glycoATP) production rates and lower mitochondrial ATP (mitoATP) production rates which is mainly from OXPHOS. Interestingly, MSCs showed enhanced glycolysis during challenge with cPE, but no significant changes in oxygen consumption rates (OCR). In accordance, seahorse assay of real-time ATP revealed glycoATP rates were elevated while mitoATP rates showed no significant differences in MSC during challenge with cPE. Furthermore, Mφ and MSCs exposed to cPE showed upregulated gene expression levels of glycolytic regulators and Mφ exposed to cPE expressed higher levels of pro-inflammatory cytokines.

**Conclusion:**

This study demonstrated the dysfunctional bioenergetic activity of bone marrow-derived Mφ and MSCs exposed to cPE, which could impair the immunoregulatory properties of cells in the bone niche. The underlying molecular defect related to disordered mitochondrial function could represent a potential therapeutic target during the resolution of inflammation.

## Introduction

Bone tissue regeneration undergoes three continuing and overlapping phases: inflammation, regeneration, and remodeling (1). While the inflammatory process is crucial for tissue repair, dysregulated inflammation, including either decreased or elevated levels, is detrimental to bone healing (2–4). Consequences of altered bone homeostasis due to dysregulated inflammation include progressive bone loss (5), impairment of bone formation and subsequent fracture healing (6, 7), implant-related osteolysis (8–10) and other problems.

Mitochondria are highly dynamic organelles that are responsible for the cell’s energy production and metabolism; they are capable of responding to the local cellular microenvironment and alter energy production and coordinate the inflammatory response of both resident and recruited cells (11). Glycolytic reprogramming is the shift from oxidative phosphorylation (OXPHOS) to glycolysis and is observed in various immune cells including macrophages (Mφ), dendritic cells, T-cells and neutrophils (12). This phenomenon is also observed in the activation of Mφ during inflammatory responses (13, 14). Pro-inflammatory Mφ demonstrate a higher level of glycolytic production of adenosine triphosphate (ATP) associated with increased production of proinflammatory cytokines including tumor necrosis factor-alpha (TNF-α), interleukin-1-beta (IL-1β), monocyte chemoattractant protein-1 (MCP-1); anti-inflammatory Mφ show a higher level of oxidative phosphorylation (OXPHOS) associated with increased secretion of anti-inflammatory cytokines such as IL-4 or IL-10. The shift in the bioenergetic pathway causes a shift in cell activation and function, resulting in a vicious cycle of disrupted cellular metabolism in the local tissues including bone (15) or muscle (16), hindering their homeostasis or regenerative potential.

Mesenchymal stem cells (MSCs) are multipotent non-hematopoietic cell precursors found in the bone marrow (17). MSCs are capable of self-renewal, multidirectional differentiation and immunoregulation, and their pluripotency makes them an ideal cell therapy for mesoderm-derived tissue regeneration and immune modulation (18, 19). Mitochondrial dynamics impact cell differentiation as well as immune function in MSCs (20). Upon osteogenic differentiation, quiescent MSCs changed their energy acquisition pathway from glycolysis to mitochondrial oxidative metabolism that generates energy through mitochondrial OXPHOS; mitochondrial dysfunction impairs this process (21–23). In addition, MSCs adapt their energetic metabolism when acquiring immunomodulatory property and shift to glycolysis (24). The immunoregulatory effect of MSCs on macrophage polarization and Th17 switch is related to the glycolytic status of the MSCs. MSCs pretreated with oligomycin decreased the M1/M2 ratio, inhibited CD4 T cell proliferation, and prevented Th17 switch to Treg cells (25). However, the role of mitochondrial bioenergetics on the immune properties of Mφ and MSCs during inflammation remains unknown.

Continuous infusion of lipopolysaccharide (LPS) contaminated polyethylene particles (cPE) has been applied to study the field of periprosthetic osteolysis and also has become a validated model for chronic inflammatory bone destruction (26–29). In order to investigate the underlying mechanisms of acute and chronic inflammatory osteolysis, we have established a cPE-induced inflammatory model using both *in vitro* and *in vivo* studies (30, 31). Previously, we have shown that cPE causes prolonged inflammation, and impaired bone formation and increased bone degradation (31). We have also shown that modulating the inflammatory status by injecting genetically modified MSCs that release the anti-inflammatory cytokine IL-4 in response to activation of the pro-inflammatory transcription factor NF-κB mitigated the above impairment in bone regeneration (32). In the current study, we used cPE to stimulate the early biological processes of the inflammatory reaction, similar to that seen to byproducts of total knee and hip arthroplasties, that eventually results in periprosthetic osteolysis. We hypothesized that inflammation would induce glycolytic reprogramming in Mφ and MSCs, associated with the release of pro-inflammatory cytokines. This *in vitro* study investigated changes in mitochondrial bioenergetics in bone marrow-derived Mφ and MSCs during challenge with cPE.

## Materials and methods

### Preparation of ultra-high molecular weight polyethylene particles

Polyethylene particles were made as previously described (33). Briefly, after washed with pure ethanol, Ceridust 3610 polyethylene particles (Clariant Corporation, CA, United States) were filtered through a 20 μm pore membrane. In the Cell Sciences Imaging Facility at Stanford University, the filtered particle size was measured as 4.62 ± 3.76 μm with a scanning electron microscope (Zeiss Sigma FESEM, Zeiss Sigma, CA, United States). After vacuum dried for 3 days, particles were resuspended in 5% BSA/PBS. The approximate concentration was tested 3.1 × 10^10^ particles/ml, and the sterility was confirmed by Limulus Amebocyte Lysate assay (Lonza, Portsmouth, NH) (34).

### Cell culture

Bone marrow-derived Mφ and MSCs were collected from C57BL/6J female mice aged 8-10 weeks old as previously described (35). Stanford Institutional Animal Care and Use Committee (IACUC) approved this isolation protocol (Protocol number: APLAC-9964) and institutional guidelines were followed and observed in all aspects. Briefly, femurs and tibias were digested under sterile conditions. Using a 25-gauge needle, the bone marrow was flushed and filtered (70 µm) into a 50 mL centrifuge tube by injecting 5 mL of macrophage basal medium (RPMI Medium 1640 supplemented with 10% fetal bovine serum, 1% Antibiotic-Antimycotic (Invitrogen, Grand Island, NY)) or 5mL of MSC basal medium (MEM alpha, supplemented with 10% FBS, 1% antibiotic-antimycotic (Thermo Fisher Scientific, Waltham, MA, USA)). Cells were centrifuged at 400 g for 10 min and resuspended in 1-3 mL/tube of iced cold red blood cell lysis buffer (Invitrogen) for 2 minutes at 4°C, followed by the addition of 20 mL/tube basal medium. Naïve macrophages were differentiated in augmented macrophage basal medium (RPMI supplemented with 10% Heat-Inactivated FBS, 1% antibiotic-antimycotic, 30% L929 conditioned medium (LCM) and 100 ng/mL M-CSF (R&D Systems, Minneapolis, MN, USA)) for 7 days (36). The attached Mφ were digested with trypsin and scraped off for further culturing or cryopreserved at -80 °C. For MSCs, cells were cultured and purified with MSC basal medium into T175 flasks until passage 6 before they were cryopreserved at -80 °C.

Mφ were seeded in the augmented basal medium in 24-well plates at a concentration of 5 x 10^5^ cells/mL with macrophage augmented basal medium with or without cPE (0.125% polyethylene particles coated added with 10 ng/ml Lipopolysaccharides (LPS, Sigma-Aldrich St Louis, MO) in 10% BSA/PBS) (37).

**Figure 1:**
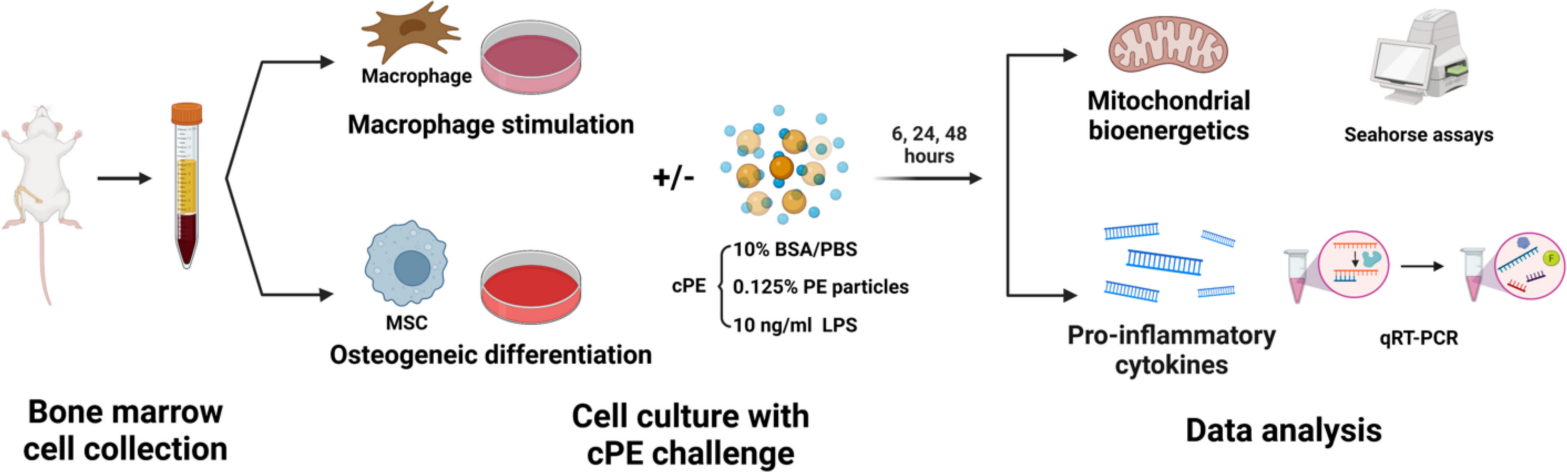
Schematic diagram of experimental design. Bone marrow-derived Mφ and MSCs were collected respectively. Cells were cultured with certain mediums for Mφ stimulation or MSC osteogenic differentiation, with or without cPE challenge. Mitochondrial bioenergetics and genes of proinflammatory cytokines were analyzed at 6, 24 and 48 hours.

P8-P10 MSCs were seeded in the basal medium in 24-well plates at a concentration of 1 x 10^5^ cells/mL overnight for attachment. Then cells were washed and cultured with osteogenic differentiation medium (α-MEM supplemented with 10% β-glycerophosphate, 50 mM ascorbic acid, 100 mM Vitamin D3, and 100 nM dexamethasone) with or without cPE. Both Mφ and MSCs samples were harvested for analysis at 4 timepoints, 0, 6, 24 and 48 hours (n = 3 per condition and per timepoint) (**Figure 1**).

### RNA extraction, cDNA synthesis and quantitative real-time PCR (qRT-PCR)

Cells from each well were lysed and collected separately using TRIzol reagent. RNA was extracted with miRNeasy kit (Qiagen, mat. No. 1071023). Purity was measured with NanoDrop One (Thermo Fisher Scientific, Waltham, MA, USA). Reverse transcribed cDNA was made by the High-Capacity cDNA Reverse Transcription Kit (Applied Biosystem, 4368814).

For quantitative real-time PCR, a reaction mix was prepared containing sample cDNA, primers, TaqMan primer-probes and TaqMan Gene Expression Master Mix (all from Applied Biosystems). Then qRT-PCR was performed on ABI 7900HT Sequencing Detection System (Applied Biosystems).

Primer sequences are summarized in **Table 1**, and β-actin was applied as in internal reference. The relative expression of target genes was calculated by the 2^−ΔΔCt^ method. All PCR reactions were conducted in triplicate.

**Table 1:** Primer sequences used in this study.

Gene name	Primer name	Primer sequence (5´ to 3´)
*Tnf-α*	Mouse _ TNF-α _F	TCTCATGCACCACCATCAAGGACT
	Mouse _ TNF-α _R	ACCACTCTCCCTTTGCAGAACTCA
*Il-1β*	Mouse_ Il-1β _F	AAGGGCTGCTTCCAAACCTTTGAC
	Mouse_ *Il-1β* _R	ATACTGCCTGCCTGAAGCTCTTGT
*Nos2*	Mouse_ *Nos2 _*F	TCTTTGACGCTCGGAACTGTAGCA
	Mouse_ *Nos2* _R	ACCTGATGTTGCCATTGTTGGTGG
*Il-6*	Mouse_ *Il-6 _*F	ATCCAGTTGCCTTCTTGGGACTGA
	Mouse_ *Il-6 _*R	TAAGCCTCCGACTTGTGAAGTGGT
*Fn1*	Mouse _ *Fn1 _*F	TGGTGGCCACTAAATACGAA
	Mouse _ *Fn1 _*R	GGAGGGCTAACATTCTCCAG
*Runx2*	Mouse _ *Runx2 _*F	CTACCCAGCCACCTTTACCTAC
	Mouse _ *Runx2 _*R	GAACTGATAGGATGCTGACGAAG
*Opn*	Mouse _ *Opn _*F	GACAACAACGGAAAGGGCAG
	Mouse _ *Opn _*R	GATCGGCACTCTCCTGGCT
*Ocn*	Mouse _ *Ocn _*F	AGGAGGGCAATAAGGTAGTGAAC
	Mouse _ *Ocn _*R	AGGCGGTCTTCAAGCCATAC
*Pfkfb3*	Mouse _ *Pfkfb3 _*F	AGAACTTCCACTCTCCCACCC
	Mouse _ *Pfkfb3 _*R	AGGGTAGTGCCCATTGTTGAA
*Hif1α*	Mouse _ *Hif1α _*F	ACCTTCATCGGAAACTCC
	Mouse _ *Hif1α _*R	CTGTTAGGCTGGGAAAAG
*Pgc-1α*	Mouse _ *Pgc-1α _*F	AAACTTGCTAGCGGTCCTCA
	Mouse _ *Pgc-1α _*R	TGGCTGGTGCCAGTAAGAG

### Analysis of cell bioenergetics

The oxygen consumption rate (OCR), the extracellular acidification rate (ECAR) and Real-Time ATP rate were measured using a Seahorse XFe96 Analyzer (Agilent, California, CA, USA) with Seahorse XF Cell Mito Stress Test Kit (Agilent, cat#: 103015-100), Seahorse XF Glycolytic Rate Assay Kit (Agilent, cat#: 103344-100) and Seahorse XF Real-Time ATP Rate Assay kit (Agilent, cat#: 103592-100), respectively, following the manufacturer’s instructions. Briefly, Mφ and MSCs were seeded in 96-well Seahorse assay plates at a concentration of 1.5 × 10^5^ or 5 × 10^4^ cells/well, respectively and cultured overnight for attachment. Prior to the assay, cells were washed, and the medium was replaced with Seahorse XF RPMI for Mφ or DMEM for MSCs supplemented with 20 mM glucose, 2 mM L-glutamine and 1 mM sodium pyruvate. Mφ and MSCs were tested separately after cPE culturing at multiple time points (6, 24 and 48 hours). Presto Blue™ assays (Thermo Fisher Scientific) were used to evaluate cell viability and normalize readings from the Seahorse XF Analyzer.

### Statistical analysis

The statistical analysis was conducted using Prism 8 (GraphPad Software, San Diego, CA). Data were expressed as mean ± Standard error of mean (SEM). Two-tailed unpaired Student’s t-test was used to compare data between groups. One-way ANOVA was used to detect differences within group between time-points. *p* < 0.05 was regarded as statistically significant.

## Results

### Reduced mitochondrial respiratory capacity in Mφ during challenge with cPE

**Figure 2:**
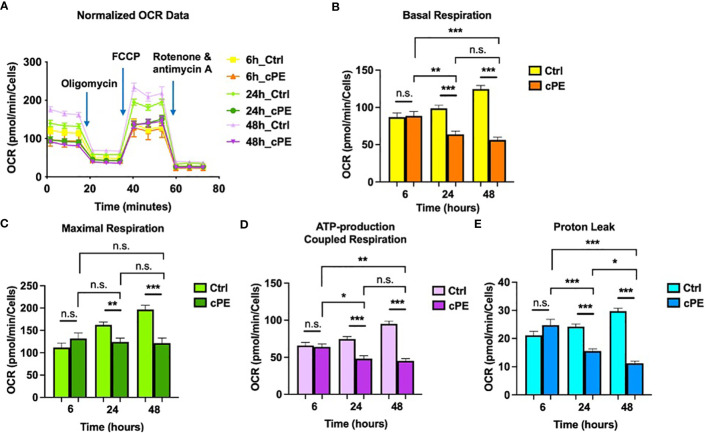
Oxygen Consumption Rates (OCR) of Mφ during challenge with cPE. **(A)** OCR of macrophages were measured with or without cPE at various time points and normalized with cell numbers with consecutive injections of oligomycin (Oligo at 1.5 μM), FCCP (1.5 μM), and Rotenone/antimycin A (0.5 μM). **(B–E)** Basal respiration, Maximal respiration, ATP-production coupled respiration and Proton leak rates were presented as bar graphs. Data are shown as mean ± SEM, ^*^ *p* < 0.05, ^**^ *p* < 0.01, ^***^ *p* < 0.001, n.s., no significance. two-tailed unpaired Student’s t-tests were compared between control and cPE groups at each time point (n = 10 per each group); One-way ANOVA was used to detect differences within group between time-points.

To determine the functional changes in the cellular metabolic activity of Mφ, we first evaluated the oxygen consumption rate (OCR; a measure of oxidative phosphorylation) using the Seahorse XF Analyzer in conjunction with the XF mito stress kit, a measure of overall mitochondrial respiration capacity. The addition of 1.5 μM oligomycin, 1.5 μM FCCP, and 0.5 μM Rotenone/Antimycin A allowed us to evaluate the contribution and sources of both mitochondrial and nonmitochondrial oxygen consumption to our measured signal. **Figure 2A** shows the calculations that can be made with the addition of each substrate. The basal respiration rates of Mφ were decreased at 24 and 48 hours of co-incubation with cPE (**Figure 2B**). The increase of oxygen consumption after adding the uncoupling agent FCCP reflected the mitochondrial reserve function. After the addition of FCCP, the maximal respiration of Mφ exposed to cPE was significantly decreased compared with the control group without cPE from 24 to 48 hours (**Figure 2C**). In addition, compared to the control group, the ATP production coupled respiration rates and proton leakage rates were reduced with significant differences in Mφ at 24 and 48 hours of challenge with cPE (**Figures 2D, E**). Our results suggested that the oxidative phosphorylation of Mφ is defective after 24 hours of challenge with cPE.

### Increased glycolysis in Mφ during challenge with cPE

**Figure 3:**
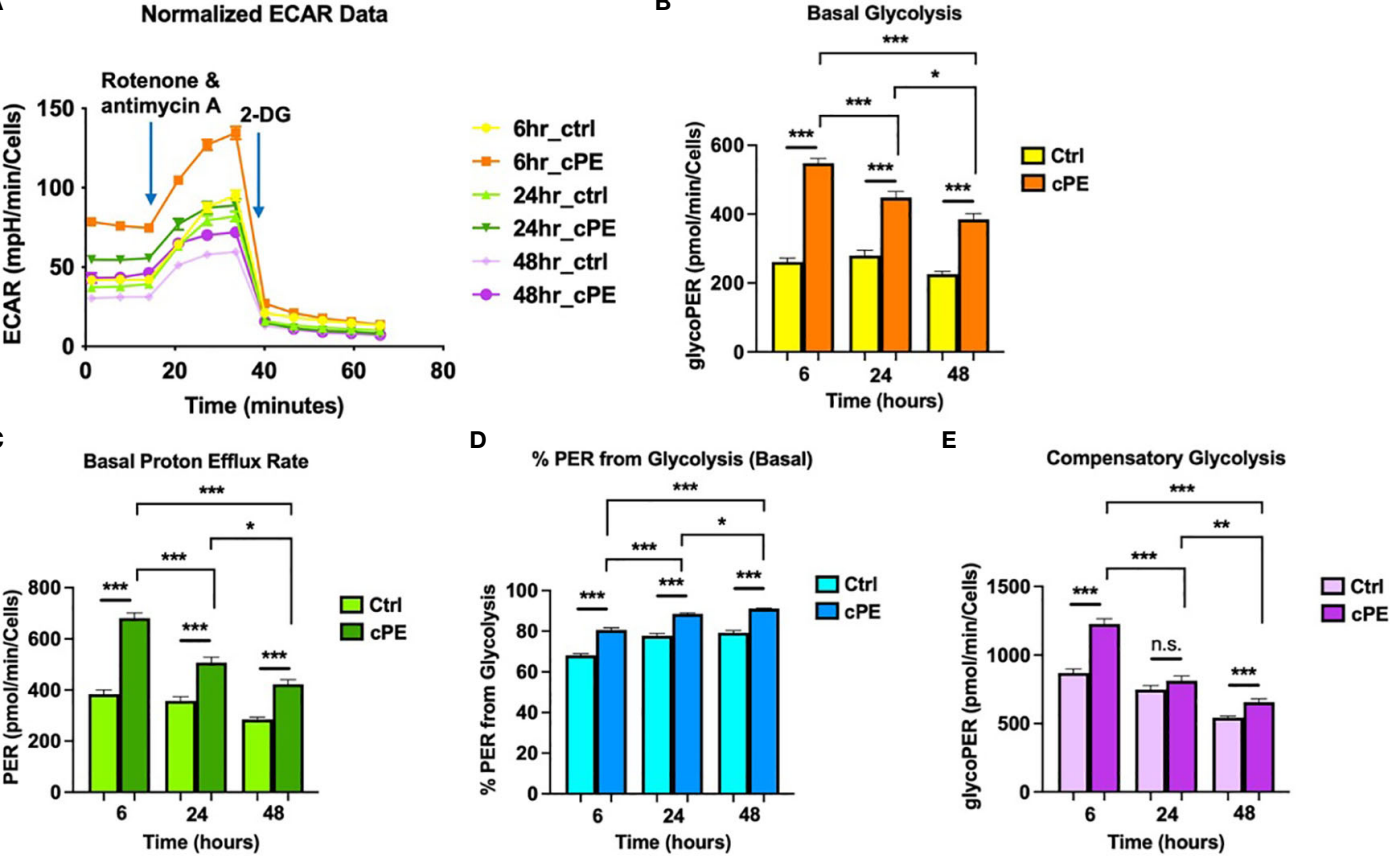
Extra Cellular Acidification Rates (ECAR) of Mφ during challenge with cPE. **(A)** ECAR of Mφ with or without cPE challenge at various time points followed by injections of Rot/AA (0.5 μM), and 2-DG (50 mM) were obtained. **(B–E)** Basal glycolysis, Basal proton efflux rate, % PER from glycolysis and Compensatory glycolysis rates were presented as bar graphs. Data are shown as mean ± SEM, ^*^ *p* < 0.05, ^**^ *p* < 0.01, ^***^ *p* < 0.001, n.s., no significance. two-tailed unpaired Student’s t-tests were compared between control and cPE groups at each time point (n =10 per each group); One-way ANOVA was used to detect differences within group between time-points.

To further investigate the mitochondrial metabolism in Mφ, we next examined the extracellular acidification rates (ECAR; a measure of glycolysis) following sequential addition of compounds containing 0.5 μM Rotenone/Antimycin A (Rot/AA) and 50 mM 2-DG (**Figure 3A**). The data showed that the basal glycolysis, physiological rate, and proton efflux rates were significantly higher starting at 6 hours in Mφ exposed to cPE compared with the control groups (**Figures 3B, C**). **Figure 3D** shows the percentage (%) of Proton Efflux Rate (PER) from glycolysis, representing the contribution of the glycolytic pathway to total extracellular acidification. The total rate of extracellular acidification is the sum of two components: respiratory acidification, in the form of CO_2_ produced in the citric acid cycle, and anaerobic glycolytic acidification in the form of lactate^-^ + H^+^ (38). We found that the %PER from glycolysis were significantly higher in Mφ challenged with cPE (**Figure 3D**). When Rot/AA was added to inhibit mitochondrial respiration, cells were able to increase energy production *via* a compensatory increase in glycolysis. This compensatory increase in glycolysis of Mφ exposed to cPE was higher than the control groups at 6 hours and 48 hours (**Figure 3E**). In conclusion, cPE exposure promoted the glycolytic pathway while suppressing mitochondrial respiration in Mφ.

### Increased production rates of glycoATP while reduced production rates of mitoATP in Mφ during challenge with cPE

**Figure 4:**
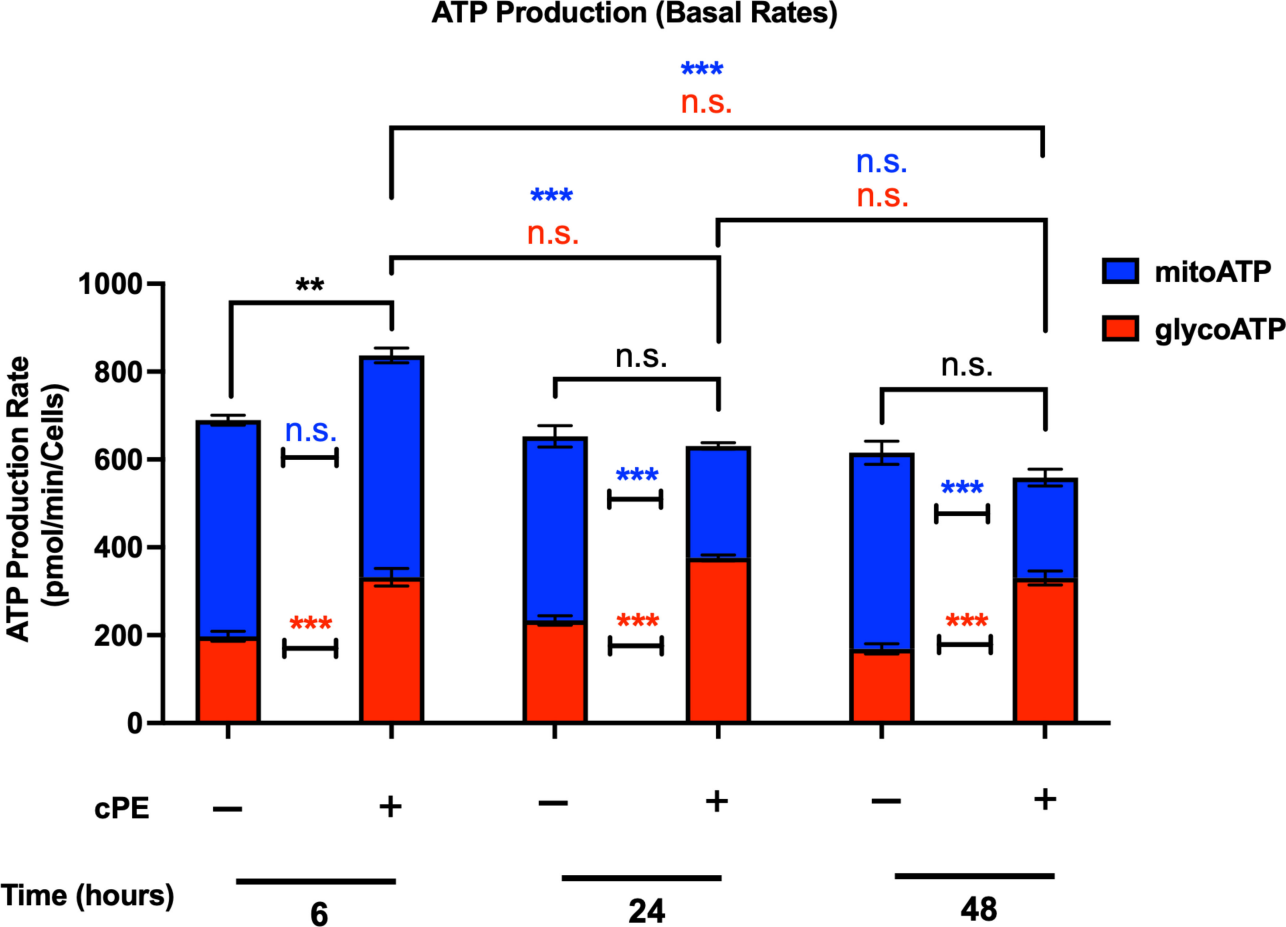
ATP production rates of Mφ during challenge with cPE. ATP production rates from mitochondrial respiration (mitoATP) and glycolysis (glycoATP) of Mφ were measured at real-time status following a sequential injection of Oligomycin (1.5 μM) and Rot/AA (0.5 μM) during challenge with cPE or not. Data are shown as mean ± SEM, ^**^ *p* < 0.01, ^***^ *p* < 0.001, n.s., no significance. two-tailed unpaired Student’s t-tests were compared between control and cPE groups at each time point (n = 10 per each group); One-way ANOVA was used to detect differences within group between time-points.

Mitochondria play a central part in cellular energy production and mediate immunomodulatory signaling. The main metabolic pathways contributing to the energy homeostasis are glycolysis and oxidative phosphorylation, which couple the breakdown of nutrients such as glucose, amino acids and fatty acids to ATP production. The real-time ATP rates were measured following a sequential injection of Oligomycin and Rot/AA using the Seahorse XFe96 Analyzer (Agilent). ATP production rates from glycolysis (glycoATP) were significantly increased in Mφ exposed to cPE at all time points (6, 24 and 48 hours), while the ATP production rates from mitochondrial respiration (mitoATP) were decreased with significance at 24 and 48 hours (**Figure 4**). The total ATP production rate of Mφ exposed to cPE was increased at 6 hours, which was mainly contributed by the glycolytic pathway as the mitoATP showed little change. These results are consistent with the OCR and ECAR results detected under the same conditions (**Figures 2**, **3**), showing that the ATP in Mφ was mainly derived from glycolysis during challenge with cPE.

### Bioenergetic profile of MSC during the challenge of cPE

**Figure 5:**
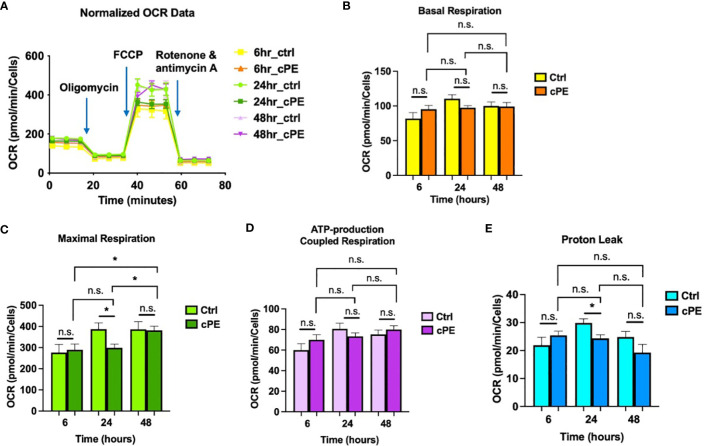
Oxygen Consumption Rates (OCR) of MSC during challenge with cPE. **(A)** OCR of MSC were measured with or without cPE at various time points and normalized with cell numbers with consecutive injections of oligomycin (Oligo at 1.5 μM), FCCP (1.5 μM), and Rotenone/antimycin A (0.5 μM). **(B–E)** Basal respiration, Maximal respiration, ATP-production coupled respiration and Proton leak rates were presented as bar graphs. Data are shown as mean ± SEM, ^*^ *p* < 0.05, n.s., no significance. two-tailed unpaired Student’s t-tests were compared between control and cPE groups at each time point (n = 5 per group of 6 or 24 hours; n = 10 per group of 48 hours); One-way ANOVA was used to detect differences within group between time-points.

The crosstalk between Mφ and MSCs plays a critical role during bone healing (39). However, the bioenergetic profile of MSCs during inflammation in bone is still obscure. To study the metabolic changes of MSC exposed to cPE, we performed the same Seahorse assays described above using Mφ. Compared to the control group, MSCs with cPE challenge exhibited little change in OCR, including basal respiration rates and ATP-production coupled respiration rates (**Figures 5A, B, D**). The maximal respiration and proton leak rates were decreased at 24 hours but showed no significant change at 6 and 48 hours in MSC exposed to cPE (**Figures 5C, E**).

**Figure 6:**
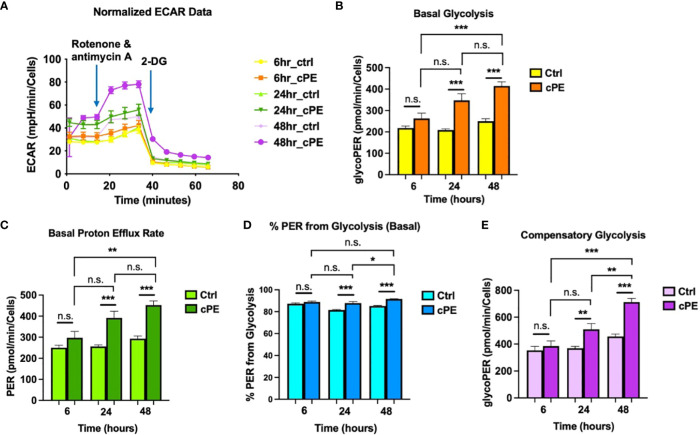
Extra Cellular Acidification Rates (ECAR) of MSC during challenge with cPE. **(A)** ECAR of MSC with or without cPE challenge at various time points followed by injections of Rot/AA (0.5 μM), and 2-DG (50 mM) were obtained. **(B–E)** Basal glycolysis, Basal proton efflux rate, % PER from glycolysis and Compensatory glycolysis rates were presented as bar graphs. Data are shown as mean ± SEM, ^*^ *p* < 0.05, ^**^ *p* < 0.01, ^***^ *p* < 0.001, n.s., no significance. two-tailed unpaired Student’s t-tests were compared between control and cPE groups at each time point (n = 10 per each group); One-way ANOVA was used to detect differences within group between time-points.

Our ECAR results revealed that the glycolytic pathway was significantly enhanced at 24 and 48 hours in MSCs during challenge with cPE (**Figure 6A**), as the basal glycolysis rates, proton efflux rates (PER) and percentage (%) PER from glycolysis were increased (**Figures 6B–D**). The compensatory glycolysis rates also showed the similar increased levels at 24 and 48 hours in MSC exposed to cPE (**Figure 6E**).

**Figure 7:**
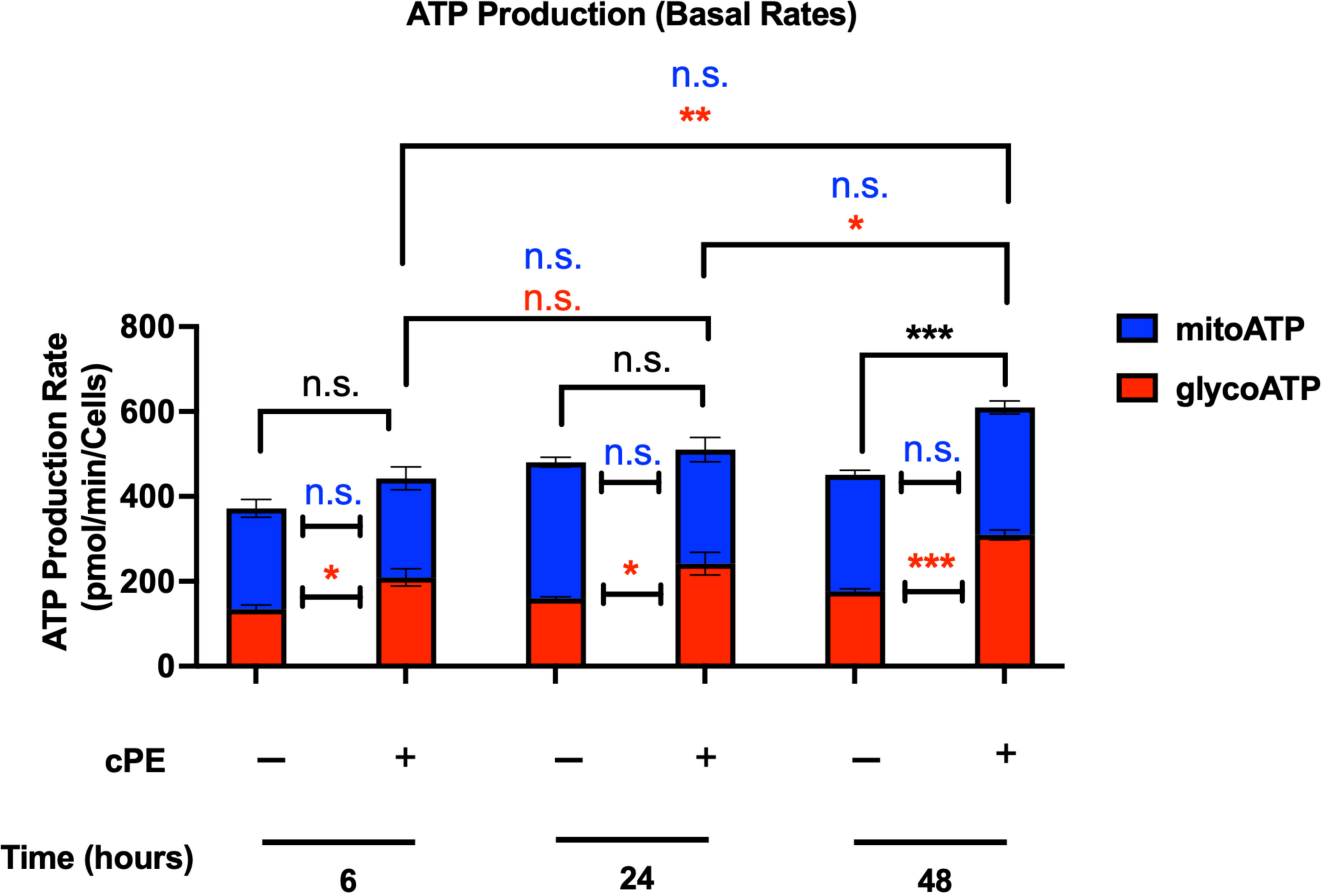
ATP production rates of MSC during challenge with cPE. ATP production rates from mitochondrial respiration (mitoATP) and glycolysis (glycoATP) of MSC were measured at real-time status following a sequential injection of Oligomycin (1.5 μM) and Rot/AA (0.5 μM) during challenge with cPE or not. Data are shown as mean ± SEM, ^*^ *p* < 0.05, ^**^ *p* < 0.01, ^***^ *p* < 0.001, n.s., no significance. two-tailed unpaired Student’s t-tests were compared between control and cPE groups at each time point (n=5 per group of 6 or 24 hours; n = 10 per group of 48 hours); One-way ANOVA was used to detect differences within group between time-points.

The glycoATP levels were significantly higher in MSCs cPE groups at all time points (6, 24 and 48 hours), and mitoATP levels exhibited little change (**Figure 7**). The total ATP level showed no significant changes at 6 and 24 hours but was increased at 48 hours in MSC with cPE challenge.

### Mφ demonstrated upregulated gene expression of pro-inflammatory cytokines after cPE challenge

**Figure 8:**
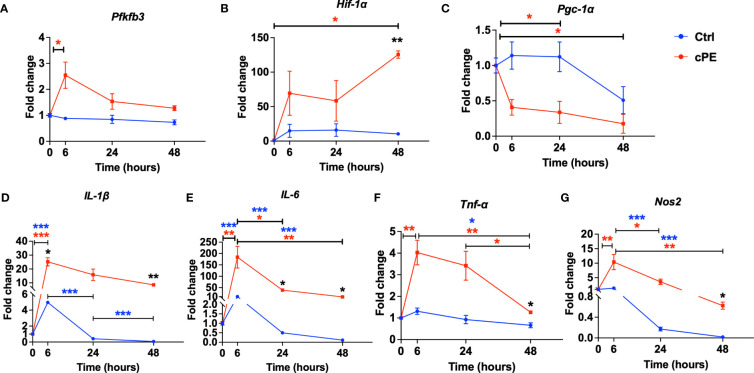
Gene expression levels of mitochondrial metabolism and pro-inflammatory markers in Mφ during cPE challenge. **(A)** RT-PCR analysis showed that compared with the control group, the expression levels of *Pfkfb3* peaked at 6 hours while the control group remained at a baseline level. **(B)** *Hif-1a* showed increased and peaked at 48 hours with a significant difference. **(C)** *Pgc-1α* showed decreased trend compared to the control group. **(D–G)** RT-PCR analysis showed that compared with the control group, the expression levels of pro-inflammatory genes *Il-1β, IL-6, Tnf-a* and *Nos2* in Mφ during cPE challenge were upregulated and peaked at 6 hours. Data are shown as mean ± SEM, ^*^ *p* < 0.05, ^**^ *p* < 0.01, ^***^ *p* < 0.001, two-tailed unpaired Student’s t-tests were compared between control and cPE groups at each time point (n=3 per each group); One-way ANOVA was used to detect differences within group between time-points.

To further investigate the metabolic and immunomodulatory changes in Mφ after cPE challenge, we quantitatively analyzed glycolysis related genes (*Pfkfb3* and *Hif-1α*) and four genes related to the immune properties of Mφ (*Il-1β, Il-6, Tnf-α and Nos2*). PFKFB3 is a critical regulatory enzyme of glycolysis, as its product Fructose 2,6-bisphosphate (F-2,6-P2), is the most potent allosteric activator of PFK-1, the second one of three rate-limiting enzymes for glycolysis (40). Glycolysis has been regarded as a cell-intrinsic property and results from the post-translational stabilization of the hypoxia-inducible transcription factor HIF-1 (41, 42). The qRT-PCR results showed that compared with the control group, the expression levels of *Pfkfb3* and *Hif-1α* were increased (**Figures 8A, B**), which is consistent with our ECAR results (**Figure 3**). Our result also showed *Pgc-1α*, which regulates mitochondrial biogenesis but also has effects on mitochondrial functions beyond biogenesis (43), showed a decrease in Mφ during challenge with cPE (**Figure 8C**). Nitric Oxide Synthase 2 (Nos2) is another marker of inflammation and enhances macrophage migration and survival (44). *Nos2* was found to be dramatically increased at 6 hours after cPE challenge (**Figure 8G**) as well as proinflammatory cytokines of Mφ (*Il-1β, Il-6, Tnf-α*) (**Figures 8D–F**). Altogether, these results suggest that cPE challenge enhanced the gene expression levels of key glycolytic enzymes and promoted the proinflammatory properties of Mφ, but impaired the gene expression of *Pgc-1α*, a mitochondrial biogenesis marker.

### MSCs demonstrated upregulation of glycolytic genes after cPE challenge

**Figure 9:**
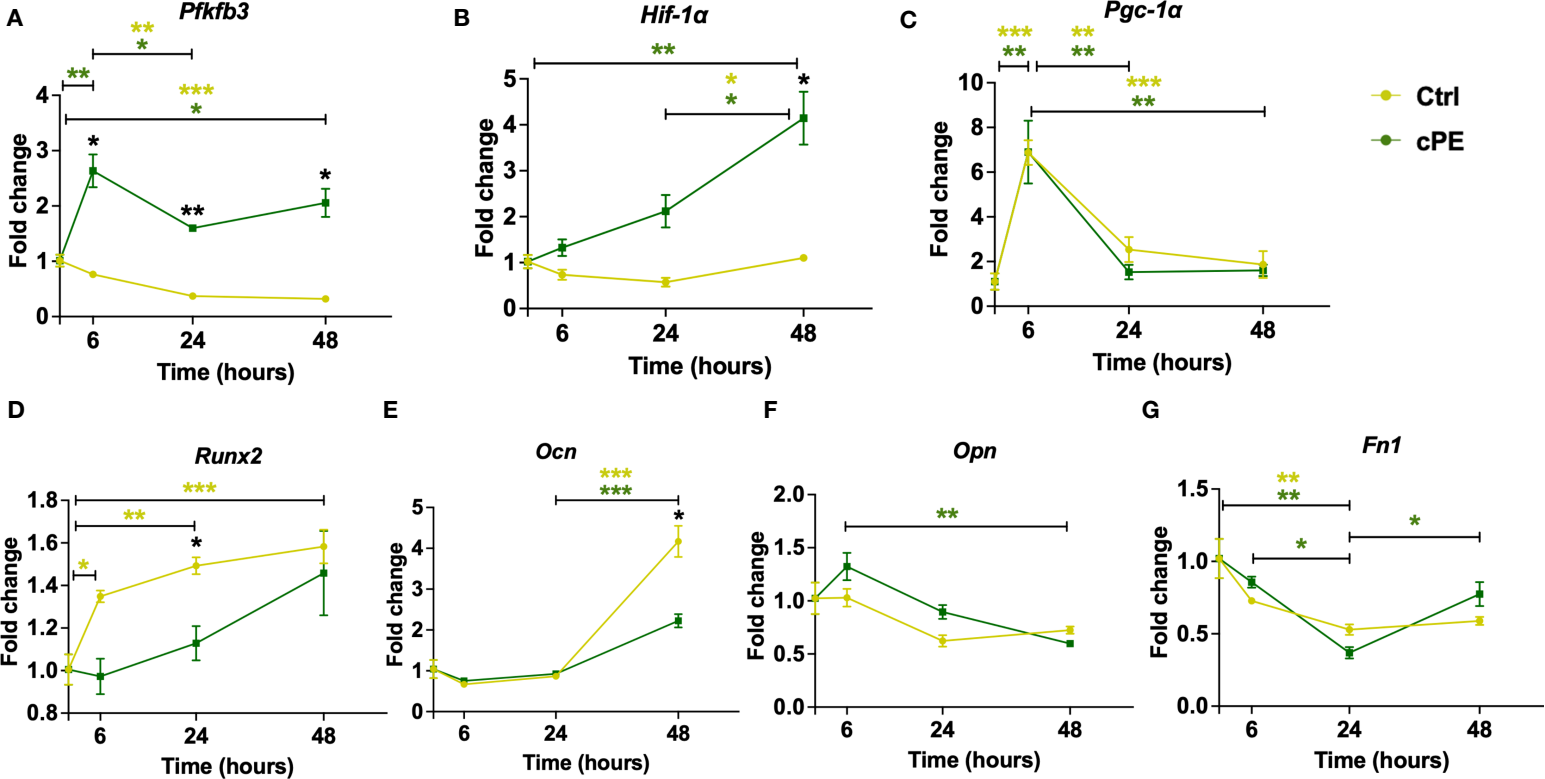
Gene expression levels of mitochondrial metabolism and differentiation markers in MSC during cPE challenge. **(A, B)** RT-PCR analysis showed that compared with the control group, the expression levels of *Pfkfb3* and *Hif-1a* were increased with significant differences. **(C)** The expression level of *Pgc-1α* showed no difference in MSC during cPE challenge compared to the control group. **(D, E)** *Runx2* and *Ocn* showed a decreased trend after cPE challenge, with a significant difference at 24 and 48 hours, respectively. **(F, G)** The expression levels of *Opn* and *Fn1* in MSC during cPE challenge showed no significant differences compared to the control group. Data are shown as mean ± SEM, ^*^ *p* < 0.05, ^**^ *p* < 0.01, ^***^ *p* < 0.001, two-tailed unpaired Student’s t-tests were compared between control and cPE groups at each time point (n=3 per each group); One-way ANOVA was used to detect differences within group between time-points.

As the mitochondrial function of MSCs closely correlate with their immunoregulatory properties and differentiation potential, we measured two genes related to mitochondrial metabolism (*Pfkfb3* and *Hif-1a*), one biogenesis marker gene *Pgc-1α* and four genes related to cell differentiation (*Runx2*, *Ocn*, *Opn* and *Fn1*) in MSCs. The expression levels of glycolytic genes *Pfkfb3* and *Hif-1α* were significantly increased in MSCs after cPE challenge compared to the control group (**Figures 9A, B**), while the mRNA levels of *Pgc-1α* showed no significant changes after cPE challenge at all timepoints (**Figure 9C**). *Runx2* and *Ocn* showed decreased levels at 24 or 48 hours, respectively in MSC exposed to cPE (**Figures 9D, E**), while *Opn* and *Fn1* were little changed at all time points (**Figures 9F, G**). These observations collectively suggested that elevated glycolysis was associated with cPE induced inflammation in MSCs.

## Discussion

Treating dysregulated inflammation-associated bone diseases remains challenging. Deciphering the metabolic reprogramming and mitochondrial dysfunction during inflammation is highly relevant to restoring the important interactions of MSCs and Mφ in the bone niche. Our goal was to determine the metabolic profile of OXPHOS and glycolysis during inflammation. Our data clearly demonstrate that bone marrow-derived Mφ and MSCs undergoing glycolytic reprogramming during inflammation induced by the challenge with cPE. Mφ have elevated glycolytic activity with decreased OXPHOS activity, while MSCs showed increased glycolysis but little change in OXPHOS. In addition, we found that Mφ and MSCs had more glycoATP but less mitoATP in cPE exposed groups compared to the control groups, which is consistent with the upregulated gene expression of glycolytic enzymes in both Mφ and MSCs. The mRNA expression level of proinflammatory cytokines (IL1-β, IL-6 and TNFα) dramatically increased in Mφ after exposure to cPE. Thus, our findings demonstrated the dynamic metabolic changes and mitochondrial function in Mφ and MSCs during early inflammation induced by the challenge of cPE.

Inflammation is an important biological response to tissue injury, and initiates and modulates the healing process. Different sets of transcription factors are activated during the inflammatory response, including the nuclear factor of the κ light chain enhancer of B cells (NF- κB), interferon regulatory factors (IRFs), signal transducers and activators of transcription (STAT) and activator protein1 (AP-1) (45). Dysregulated inflammation is associated with many skeletal diseases, such as osteolysis, non-union of fractures, and osteoarthritis. To decrease the intensity of persistent inflammation, it is possible to regulate the signaling pathways of proinflammatory transcription factors. NF-κB regulates the synthesis and expression of numerous proinflammatory molecules. We showed that suppression of NF-κB signaling mitigates polyethylene wear particle-induced inflammatory response (46–50). Although mitochondria are the major organelles for energy production, they also are intimately related to the inflammatory response. For example, specific metabolic pathways are increasingly being recognized as critical hallmarks of macrophage subsets: inflammatory M1 Mφ display enhanced glycolytic metabolism and reduced mitochondrial activity (51). Conversely, anti-inflammatory M2 Mφ show high mitochondrial oxidative phosphorylation (OXPHOS) and are characterized by an enhanced spare respiratory capacity (SRC). Previous studies have demonstrated that oxygen deprivation induced an increase in HIF-1α associated with the activation of glycolytic pathways, leading to an M1 polarization (52). Therefore, our findings of enhanced glycolysis (**Figure 3**) and increased *Pfkfb3* and *Hif-1α* levels (**Figures 8A, B**) imply that Mφ are polarized to M1 during the challenge of cPE. Moreover, the mitochondrial function can impact innate immunity by affecting the transcriptional regulation of inflammatory cytokines, *via* NF-κB and HIF-1α target inflammatory genes and antiviral type I IFNs genes (53). Accordingly, our real-time qPCR results (**Figures 8D–G**) showed that the proinflammatory cytokines (IL-1β, IL-6 and TNFα) and the pro-M1 gene Nos2 were increased at the molecular level, which could be the consequence of activated NF-κB and HIF-1α pathways induced by the challenge of cPE. M1 macrophage-related cytokines such as TNF-α, IL-6 and IL-1β inhibit osteogenesis and induce osteoclastogenesis (54, 55), whereas the M2 macrophage-related cytokines such as IL-4 and IL-10 have the opposite properties and inhibit osteoclastogenesis through the inhibition of NFATc1 (56, 57). This suggests that glycolytic reprogramming leads Mφ to an M1 phenotype and increased proinflammatory cytokines (IL-1β, IL-6 and TNFα) production, which in turn contribute to impaired osteogenesis.

Mitochondrial dysfunction in tissue-specific MSCs plays a critical role in cell fate and the morbidity of acute or chronic inflammation-associated bone diseases. As the most important cells in bone formation and remodeling, tissue resident MSCs were affected by inflammation in every respect, including proliferation, migration, and differentiation (58). Murphy et al. demonstrated that MSCs isolated from patients with end-stage osteoarthritis (OA) are functionally deficient in terms of *in vitro* proliferation and differentiation (59). Interestingly, a recent study also identified that chronic inflammation contributes to defective osteogenic differentiation in MSCs by impairing endoplasmic reticulum (ER) function, prolonging ER stress and inducing MORF-mediated-PERK transcription (60). However, the effects of mitochondrial dysfunction on the immunomodulatory properties of MSCs have not been fully determined. Thus, understanding the bioenergetic and immunological features of MSCs is critical to develop and optimize MSCs-based therapies. In the current study, we investigated the bioenergetic profile and mitochondrial function of MSCs during inflammation induced by cPE. The elevated ECAR with stable OCR in MSCs after cPE challenge (**Figures 5**, **6**) elucidated the metabolic switch and functional adaptions biologically, which further impact the generated immune response and inflammatory status in the context of cell stress.

The question remains of how ECAR is enhanced in MSCs during the challenge of cPE. MSCs are the most promising stem cells for the treatment of inflammatory and immune diseases due to their inherent immunomodulatory properties and self-renewal abilities (61). In addition to the regulation of proliferation and differentiation, glycolysis is reported to facilitate and maintain MSC immune function (24). As a signaling molecule regulating glycolysis-associated immunomodulation, Hif-1α played a pivotal role in MSC immunomodulation and metabolism (25, 62, 63). Hif-1α overexpressed MSCs promoted expressions of immunosuppressive factors (IDO, COX2 and PD-L1) and secreted more extracellular vesicles (EVs), with better anti-inflammatory therapeutic effects in macrophage polarization (64). Besides, a previous study suggested that HIF-1α engaged a transcriptional paradigm that shifts cellular bioenergetics toward anaerobic glycolysis (65). In line with this finding, our qPCR data showed that the gene expression levels of HIF-1α were significantly increased in MSCs after cPE exposure. Thus, the activation of HIF-1α regulatory pathway could be a potential driver in the glycolytic reprogramming to promote MSC’s immune function during the challenge of cPE.

Furthermore, Shum et al. demonstrated HIF-1a as a key regulator of MSC energy metabolism during the osteogenic differentiation (22). While it is dramatically downregulated in osteogenic MSCs, reactivation of HIF-1a led to a significant glycolytic reprogramming and decreased ALP during differentiation. In addition, they found no difference between osteogenic MSCs and the control group regarding the OCR and ECAR index until 14 days later. Our OCR and mitoATP remained stable in both MSCs groups within 48 hours (**Figures 5**, **7**). The qPCR data showed that *Runx2* and *Ocn* was significantly decreased with cPE challenge (**Figures 9D, E**), while other osteogenic gene markers had little changes (**Figures 9F, G**). This may be due to the early analysis time for differentiation. Taken together, our findings implied that the challenge of cPE resulted in a preconditioned MSC status with enhanced immune functions, which may compromise the osteogenesis differentiation. The primary genes involved in leading mitochondrial dysfunction during the chronic inflammation at longer time points need to be further revealed in order to provide more insight for future treatment of chronic inflammatory bone healing.

The transcriptional co-activator peroxisome proliferator-activated receptor gamma coactivator 1 alpha (PGC-1α) regulates mitochondrial biogenesis and functions including fission, fusion, and mitophagy. PGC-1α has also been characterized as a major factor in the cellular energy metabolism (66). PGC-1α stimulates mitochondrial biogenesis and promotes the remodeling of metabolically more oxidative and less glycolytic energy production, and participates in the regulation of both carbohydrate and lipid metabolism. Our qPCR data showed the gene expression level of *Pgc-1α* was decreased in Mφ exposed to cPE (**Figure 8C**), which is consistent with increased glycolysis and low oxidative phosphorylation as previously reported. This finding suggested that PGC-1α might be a therapeutic candidate of Mφ for related inflammatory bone disease. Intriguingly, different from Mφ, our qPCR data of *Pgc-1α* in MSC showed no significant changes during the cPE challenge (**Figure 9C**). It was reported that PGC-1α was increased in the osteogenically differentiated MSCs and peaked on the 7th day of induction in comparison with undifferentiated MSCs (67). Moreover, the *in vivo* and *in vitro* experiments showed that deleting PGC-1α suppresses differentiation and activity of osteoblasts, resulting in a significant decrease of cortical thickness and trabecular thickness (67, 68). Our qPCR results showed unchanged mRNA expression levels of *Pgc-1α* (**Figure 9C**), coupled with the decreased gene expression levels of osteogenic differentiation genes (*Runx2* and *Ocn*) in MSCs exposed to cPE (**Figures 9D, E**). This may imply that cPE impaired MSC-mediated osteoblastogenesis.

The crosstalk between Mφ and MSCs plays an important role in the development of skeletal diseases, as well as the maintenance of homeostasis of inflammatory microenvironments. For example, successful fracture healing depends on the coordinated cross-talk between Mφ and MSCs (39, 69). Once the fractures and injured tissue triggered the recruitment of monocytes/macrophages, activated inflammatory M1-like Mφ were dominant in the early stage to clear cell debris and pathogens *via* pro-inflammatory cytokine release, contributing further to bone regeneration. At the later stages, polarized M2 Mφ with secreted IL-4 and IL-10 can facilitate bone formation through MSC-mediated osteogenesis, reflecting an essential supporting role of M2 Mφ in fracture healing (70). On the other hand, MSCs regulate the function of Mφ through their immunomodulatory functions in response to an inflammatory microenvironment (71, 72). However, the exact mechanism of Mφ-MSC crosstalk in bone healing remains to be determined, particularly under inflammatory microenvironments.

In the present study, we studied Mφ and MSCs separately to determine whether the observed metabolic reprogramming was due to macrophages or MSCs individually. Studying these 2 cell types together in co-culture using the Seahorse Assay would not discriminate which specific cell type might be responsible for the observed findings. Interestingly, we found that both macrophages and MSCs exhibit glycolytic reprogramming. Thus, it follows that co-cultures of these cells would demonstrate the same phenomenon. In fact, we have performed this co-culture experiment using a 3D model (30). The results confirmed that glycolytic reprogramming does indeed occur in co-culture of MSCs and Mφ using comparable techniques including PCR.

There are still several limitations to this study. Firstly, we investigated this *in vitro* study with cPE-induced inflammatory microenvironment based on our well-established chronic inflammatory bone loss mouse model (32). Longer term analysis is further needed to explore the overall changes regarding osteogenesis and immunomodulation with cPE challenge. Secondly, further analysis is necessary to demonstrate the molecular mechanisms of glycolytic reprogramming and its roles in regulating immunomodulatory properties during inflammation.

## Conclusion

We demonstrated the dynamic bioenergetic profiles in Mφ and MSCs, respectively co-incubated with or without cPE. The Seahorse bioenergetic data showed elevated glycolysis and decreased OXPHOS in Mφ, while MSCs exposed to cPE showed enhanced glycolysis but unchanged level of OCR. Similarly, upregulated glycolysis related genes *Pfkfb3* and *Hif-1α* were detected in Mφ and MSCs exposed to cPE. Furthermore, our qPCR data revealed cPE enhanced the gene expression of proinflammatory cytokines and inflammation markers, including *Il-1β, Il-6, Tnf-α and Nos2* in Mφ. Differentiation related genes *Runx2* and *Ocn* showed decreased gene expression levels in MSC during challenge of cPE.

## Data availability statement

The original contributions presented in the study are included in the article/supplementary material. Further inquiries can be directed to the corresponding authors.

## Ethics statement

The animal study was reviewed and approved by Stanford’s Administrative Panel on Laboratory Animal Care (APLAC).

## Author contributions

Designed experiments: XL, HS, MZ, QG, SG. Performed experiments: XL, HS, MZ, VT, EH. Data analysis: XL, HS, MZ, NZ. Writing and editing of manuscript: XL, HS, NZ, SC, SG, MZ, VT, QG, MTs, MTo, JK, EH, CM, CC. All authors contributed to the article and approved the submitted version.
